# Gene expression profiling of tumor-initiating stem cells from mouse Krebs-2 carcinoma using a novel marker of poorly differentiated cells

**DOI:** 10.18632/oncotarget.14116

**Published:** 2016-12-23

**Authors:** Ekaterina A. Potter, Evgenia V. Dolgova, Anastasia S. Proskurina, Yaroslav R. Efremov, Alexandra M. Minkevich, Aleksey S. Rozanov, Sergey E. Peltek, Valeriy P. Nikolin, Nelly A. Popova, Igor A. Seledtsov, Vladimir V. Molodtsov, Evgeniy L Zavyalov, Oleg S. Taranov, Sergey I. Baiborodin, Alexander A. Ostanin, Elena R. Chernykh, Nikolay A. Kolchanov, Sergey S. Bogachev

**Affiliations:** ^1^ Institute of Cytology and Genetics, Siberian Branch of the Russian Academy of Sciences, Novosibirsk 630090, Russia; ^2^ Novosibirsk State University, Novosibirsk 630090, Russia; ^3^ Softberry Inc., New York 10549, USA; ^4^ The State Research Center of Virology and Biotechnology VECTOR, Koltsovo, Novosibirsk 630559, Russia; ^5^ Institute of Clinical Immunology, Siberian Branch of the Russian Academy of Medical Sciences, Novosibirsk 630099, Russia

**Keywords:** tumor-initiating stem cells, DNA internalization, RNAseq, Real Time PCR, TAMRA

## Abstract

Using the ability of poorly differentiated cells to natively internalize fragments of extracellular double-stranded DNA as a marker, we isolated a tumorigenic subpopulation present in Krebs-2 ascites that demonstrated the features of tumor-inducing cancer stem cells. Having combined TAMRA-labeled DNA probe and the power of RNA-seq technology, we identified a set of 168 genes specifically expressed in TAMRA-positive cells (tumor-initiating stem cells), these genes remaining silent in TAMRA-negative cancer cells. TAMRA+ cells displayed gene expression signatures characteristic of both stem cells and cancer cells. The observed expression differences between TAMRA+ and TAMRA− cells were validated by Real Time PCR. The results obtained corroborated the biological data that TAMRA+ murine Krebs-2 tumor cells are tumor-initiating stem cells. The approach developed can be applied to profile any poorly differentiated cell types that are capable of immanent internalization of double-stranded DNA.

## INTRODUCTION

Tumor-initiating stem cells (TISCs) were discovered in the late 1990ies [1, 2]. They have a set of specific properties that are shared between stem and cancer cells. These cells are capable of self-renewal; they maintain the pluripotency state and determine the tumorigenic potential of a graft. One of the major features of TISCs is their ability to form metastases, accounted for by the change in the expression of the genetic network controlled by the epithelial-mesenchymal transition [3].

Understanding the molecular basis of TISC functioning and development of approaches to their eradication are related to two practical tasks: identification of these cells in a pool of cancer cells and establishing a molecular portrait of gene expression in these cells. There are several ways of identifying TISCs in the pool of cancer cells [4–9]. One of them is based on the analysis of cell surface markers specific for this type of cells [6]. To date, no universal cell surface marker has been identified that could be used to identify TISCs in different cancers. Another way of detecting TISCs is based on their ability to efflux lipophilic dyes, such as rhodamine or Hoechst. It is these two methods that are used for isolating TISCs in the numbers required for conducting either microarray or RNAseq analyses and for retrieving the molecular portrait of this type of cells. Using these two approaches, molecular portraits of gene expression of several types of TISCs were created [9–15].

In our studies [16–20], we characterized a new marker of poorly differentiated cells of different origin, including TISCs, – the ability to internalize fragments of extracellular double-stranded DNA (such as plasmid DNA or FITC/TAMRA-labeled PCR products) by a natural internalization mechanism.

It was shown on the model of Krebs-2 ascites tumor that TAMRA+ cells trigger growth of a new tumor with similar histological characteristics. Elimination of these cells results in elimination of the engraftment potential of the cells and in curing of the mice from developed Krebs-2 ascites [16, 20].

In this study, we used the above marker to identify a set of genes overexpressed in TAMRA+ Krebs-2 cells. Based on this, a molecular portrait of gene expression in TAMRA+ Krebs-2 tumor cells was obtained, which firmly positions these cells as TISCs.

## RESULTS

### Internalization of TAMRA-labeled DNA fragments by Krebs-2 cells, RNAseq

**Figure 1:**
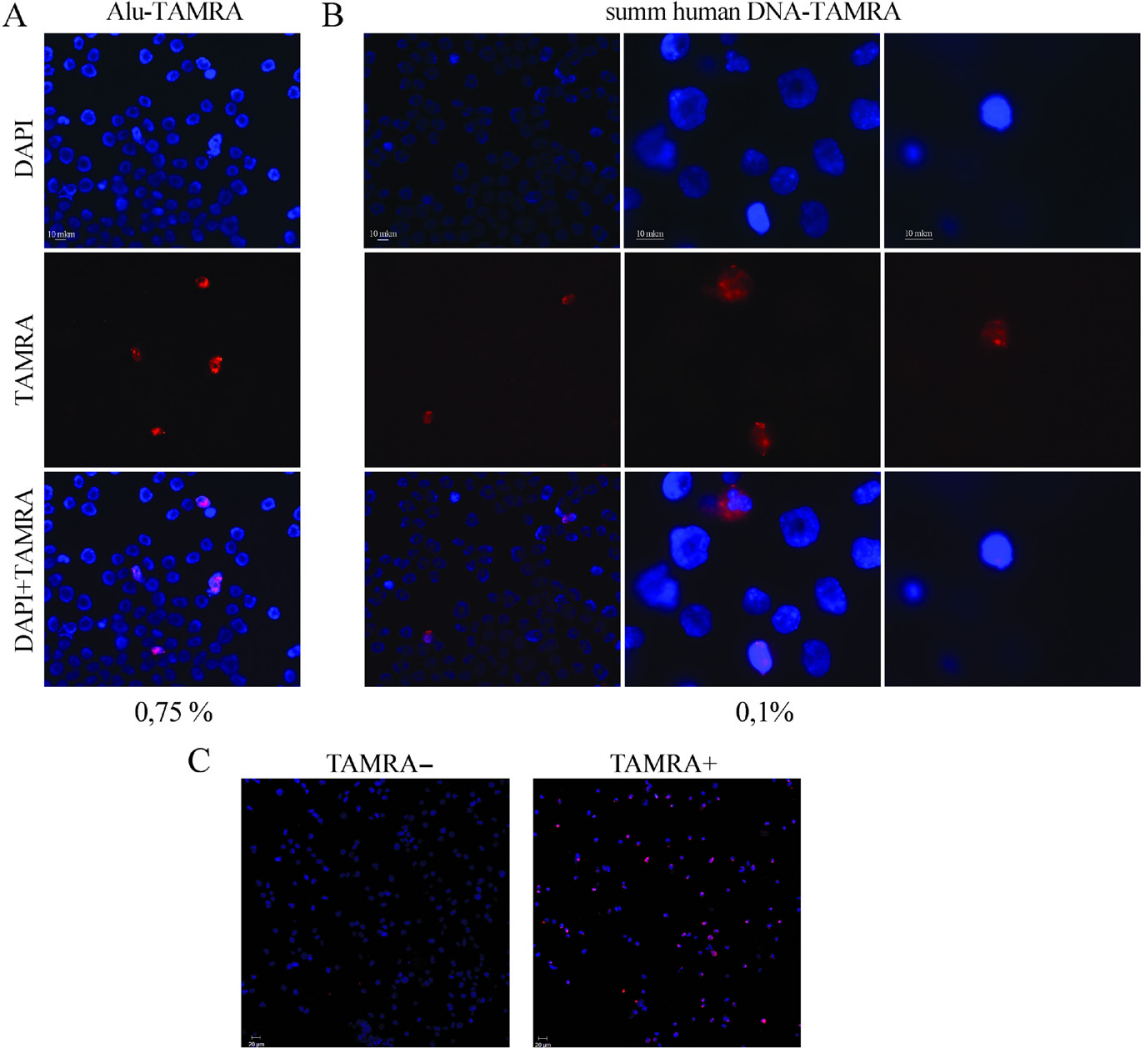
Analysis of internalization of a TAMRA-labeled DNA into the Krebs-2 cells (Axioskop 2 Plus, Zeiss) (**A**) Internalization of a TAMRA-labeled human *Alu*-fragment into the ascites Krebs-2 cells. (**B**) Internalization of TAMRA-labeled total fragmented human DNA by Krebs-2 ascites. (**C**) Analysis of enrichment of the population TAMRA– and TAMRA+ Krebs-2 cells by the sorting procedure (BD FACSAria, Becton Dickinson).

It has been previously demonstrated that Krebs-2 ascites cells internalize TAMRA-labeled DNA probe by a natural internalization mechanism [16, 20]. 0.1 to 3% of Krebs-2 cells are naturally capable of capturing extracellular double-stranded DNA fragments (Figure 1A, 1B). Using this property, the populations of cells containing TAMRA-labeled DNA and TAMRA– cells were isolated from TAMRA-DNA treated Krebs-2 cells by repeated sorting (BD FACSAria, Becton Dickinson), with TAMRA+ target cells becoming 50% to 80% enriched and with nearly 100% pure TAMRA – cells (Figure 1C).

To obtain and analyze RNA transcripts, the RNAseq was applied. As a result, 2 million reads were obtained.

### Trimming, analysis and mapping of reads

**Figure 2:**
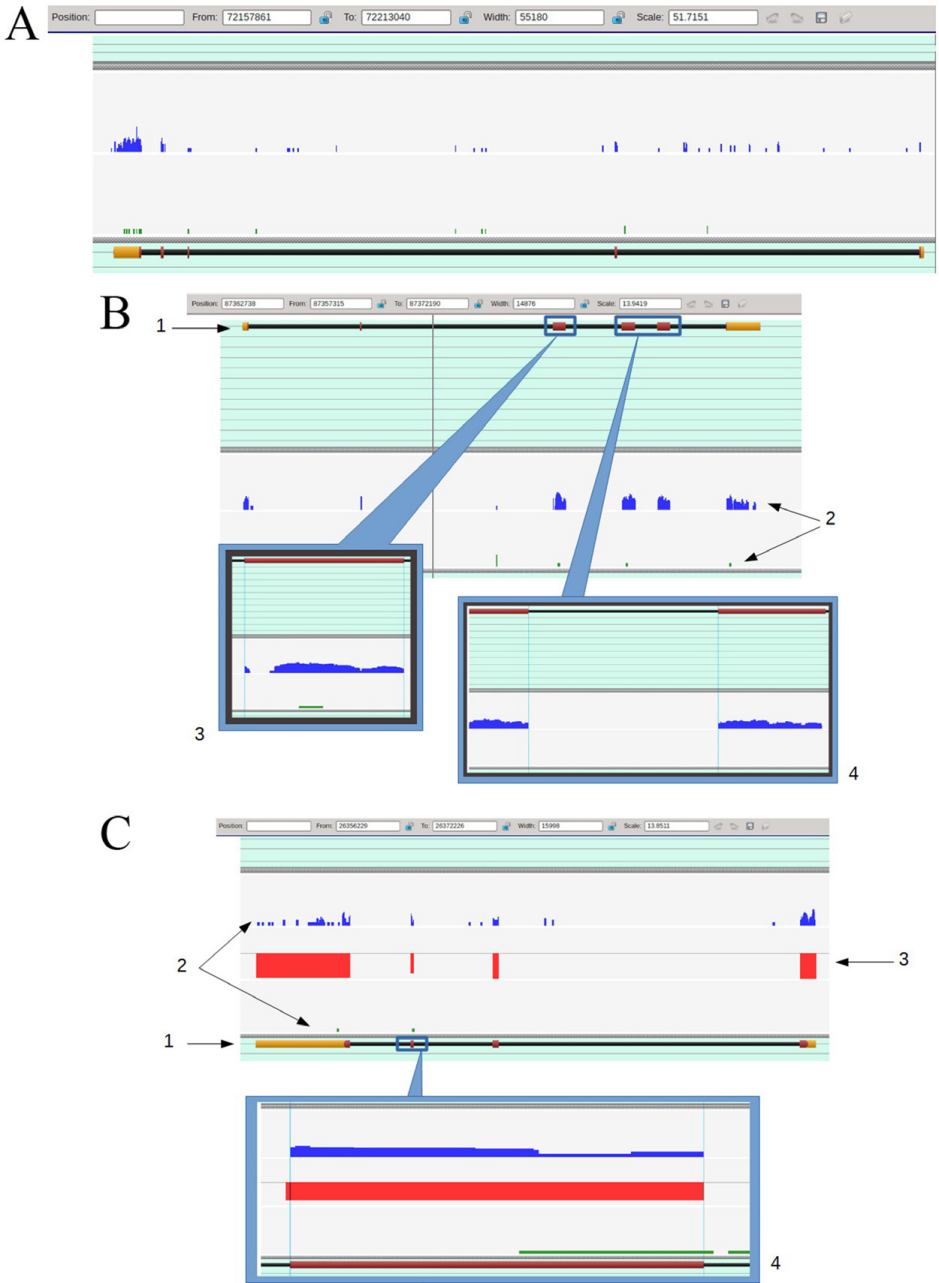
(**A**) The general view of the expression profiles for TAMRA+ (blue profile) and TAMRA– (green profile) cells overlaid to melanoregulin gene (*Mreg*, uc007bkg.2, chromosome 1, – strand) marking. The gene consists of 5 exons visualized as yellow and dark purple filled rectangles. The yellow color corresponds to non-coding (untranslatable) sequences, the dark purple one – to coding sequences. Both profiles contain peaks that lay inside the boundaries of the gene, but in the case of TAMRA– cells, most of them are located over introns and therefore present no interest for us, while the TAMRA+ profile contains the peaks exactly corresponding to exons. (**B**) The detailed view of expression profiles for TAMRA+ (blue profile) and TAMRA– (green profile) cells overlaid to *Cd5l* gene (*Cd5l*, uc008psa.2, chromosome 3, + strand) marking. 1 – Gene layout. The gene consists of 6 exons that are drawn as filled rectangles over the black line, which in turn represents the gene's location over the chromosome. The yellow color corresponds to non-coding sequences, the dark purple– to coding sequences. Exons are counted from left to right. 2– Expression profiles for TAMRA+ (the upper blue profile) and TAMRA – (the lower green profile). The peaks height represents the number of reads dropped to a certain chromosome region. 3 – Detailed view of exon 3. It is demonstrative that despite the gap inside the exon's boundaries (marked by vertical blue lines), the short tails of the reads were aligned precisely to the exon layout. TAMRA– profile also contains a single read that dropped to this exon. We cannot determine whether this event is accidental or not, since it cannot be discriminated by nucleotide mismatching or supported by intronic evidence. 4 – Detailed view of exon 4 to exon 5 junction. It is demonstrative that “after splicing” the expression profile would be smooth and continuous. (**C**) The detailed view of expression profiles for TAMRA+ (the blue profile) and TAMRA– (the green profile) cells overlaid to Claudin 1 gene (*Cldn1*, uc007yuz.2, chromosome 16, – strand) marking. 1 – Gene layout. 2 – Expression profiles. 3 – “Contrasting bar”. This “profile”, which consists of red bars representing the difference between two expression profiles exactly inside exons boundaries, is used just for greater convenience in the visual analysis. The higher the bar, the more significant the difference is. The absolute difference (one of profiles contains no reads dropped to a certain exon) is represented by a full bar and corresponds to 100%. The direction of a bar (up or down) displays the profile with a lower expression level. It can be seen that TAMRA – cells profile demonstrates almost absolute lack of expression. 4 – Detailed view of exon 3. The TAMRA– profile contains a single read dropped to exon but its right tail lays outside the exon's boundary, which means the accidental nature of this event. In fact, we have an absolute difference between TAMRA+ and TAMRA– profiles, but the viewer had drawn a limited difference bar due to “formal hit” in TAMRA– profile.

Figure 2 shows representative snapshots and illustrates the general approach to visual analysis of mapping data that will be further referred to as “expression profiles”. Since it was practically impossible to isolate the pure population of TAMRA + cells in the amount sufficient for RNA isolation and subsequent RNA sequencing (10- to 12-fold enrichment of this population could only be achieved; additional sorting rounds drastically decreased the cell survival and, as a consequence, RNA availability), while the pure TAMRA – population was available almost unlimitedly, we selected only those genes which were exclusively expressed in TAMRA+ cells.

Following primary visual inspection, we obtained a set of approximately 300 genes. For these genes, we retrieved the appropriate mRNA sequences, which were further used as target sequences for re-mapping of the total reads, both from TAMRA+ and TAMRA– datasets. In total, 3 mapping rounds were performed with sequentially more stringent specificity thresholds, which allowed us to filter out a significant number of genes that did not pass the test. This step was necessary due to a small number (2 millions) of reads obtained, which in turn was the consequence of previously described difficulties. Such a small number of mapped reads is supposed to produce a number of “homeless” reads, which can occasionally be mapped to the wrong place, and thus give a false positive or false negative signal.

As a result, we obtained a set of 168 genes that were expressed in TAMRA+ cells and not expressed (or expressed at an extremely low level) in TAMRA– cells. Further, these genes were subjected to qPCR analysis for additional verification.

### Systemization of genes and their breakdown into functional groups

Supplementary Table S1 contains a list of 168 genes with their GenBank accession numbers, the frequency of reads in TAMRA– and TAMRA+ cells, and qPCR expression data for select genes (TAMRA+ vs TAMRA– cells). In addition, for some genes the information on the type of protein and subcellular localization in accordance with the classification of the *Ingenuity Pathway* Analysis (IPA) is provided.

To determine whether a gene is involved into molecular pathways determining the basic biological features of a cell, two different approaches were used.

The first approach was based on the data obtained in the study of Dolgova et al. [16], which indicated that TAMRA+ cells possessed the features of TISCs. This in turn indicated that gene ontology (GO) terms related to “stemness” and “cancer” should be overrepresented among the genes specific for TAMRA+ cells. Whether this was indeed the case was tested by matching their properties characterized in the original papers against the above GO categories.

### “Stemness” genes

A stem cell is characterized by two features: the ability to divide asymmetrically and the ability to develop into any types of cells of the body (the pluripotency feature), transmitting this property to one of the daughter cells throughout many acts of cell division.

Asymmetric division of the stem cells is ensured by the HH, NOTCH and WNT pathways [21–27]. The pluripotent status of the stem cells is primarily maintained via retinol signaling system [28]. Thus, to test the “stemness” the genes specifically expressed in TAMRA+ cells, they were considered in terms of their participation in pluripotency maintenance and asymmetric division.

Asymmetric division (group one) – *Asb4, Nfatc2, Ppap2b, Tcf7l2, Uty, Wnt5a, Dnaja4, Rab37, Thpo, Sox9*.

Pluripotency maintenance (the molecular factors of the regulation system of retinol) (group two) – *Zfp39, Abca1, Abca9, Abca13, Aldh1a1, Aldh1l1, Bmper, Crabp2, Cyp2d26, Cyp26a1, Eif2s3y, Igf1, Igf2, Pik3ip1, Rragd, Rbp7, Rrh*.

Other factors of stem cells (group three) – *Clec11a, Lhx4, Gtf2a1l, Tbata, Mreg, Slc43a3, Ifi27l2b*.

Altogether, 34 genes were found in the group of genes determining the “stemness” properties of the TAMRA+ cells. The ability of cells to undergo asymmetric cell division is characterized by the activity of the genes of the WNT-dependent signaling cascade (10 genes). Maintenance of the pluripotent state throughout numerous divisions is determined by the signaling cascade of the retinol system (17 genes). There are genes in each group that are known as key regulators of these cascades. For group one, these include the transcription factors *Sox9*, *Nfatc2*, *Tcf7l2*, *Ppap2b*, and secreted cytokines *Wnt5a*, *Thpo*. For group two, these are growth factors *Igf1* and *Igf2*. Besides, TAMRA+ cells are characterized by the expression of genes belonging to other molecular systems of stem cells (group three). These are the growth factor *Clec11a*, the transcription factor *Lhx4*, and the proteins *Gtf2a1l*, *Tbata*, *Mreg*, *Slc43a3*, *Ifi27l2b*.

### “Cancer” group genes

TISCs possess the following unique properties allowing them not to depend on morphogenetic laws determining the orderliness of the cell systems of a body. When dividing, TISCs form two types of cells. A cell is formed, completely identical with the original totipotent cells, plus a committed cell, capable of multiple, though not infinite, division. A TISC is able to induce development of a new tumor characterized by the same histological features as the original tumor [29–31]. Further, TISCs are capable of metastasizing [32–34]. The ability of TISCs to metastasize is determined by the property of these cells to undergo epithelial-mesenchymal transition [35–39]. Remodeling of the cytoskeleton occurs, and factors altering the structure of the extracellular matrix in the cellular environment become expressed. A cell is thus able to cross the basal membrane and the endothelial wall, to move with the blood flow, to extravasate, and to form a new tumor nest. In this process, a TISC consistently activates the respective gene systems, acquires the property of escaping the attack of the immune cell system [40] and activates antiapoptotic genes [41–44]. In addition, TISCs possess other unique qualities, such as the ability to eliminate and neutralize toxins [45–49], to interact with the cells of the surrounding stroma and to stimulate them to transition to anaerobic respiration (glycolysis), which is known as the Warburg effect [50–52]. Regulation of growth and differentiation of a tumor cell is also related to metabolism of Ca2+ and is determined by calcium-dependent protein kinase (PKC). Normally, PKC functions as a modulator and balances the growth and differentiation processes. Tumor cells are invariably characterized by PKC overactivation, which in turn induces proliferation, stimulates formation of phosphotyrosine, and enhances uncontrolled multiplication of cells. PKC is known to be activated by 3-phosphoinositol-dependent protein kinase (PDK) [53–55]. In addition, apoptotic blebs of cancer cells were described to fuse together around the main body to form so-called blebbishields that have the properties of a TISCs [56]. Each of the above properties is characterized by concerted activity of a certain set of genes. Just as above, the selected gene set was analyzed for possible involvement into cancer based on the literature data.

Oncogenes, oncosuppressors – *Eef1a2, Per2, Rasgrp3, Sash1, Tal1*.

Genes characteristic of cancer cells – *Oit3, Tnxb, Zfr2, VpreB3, Trim40*.

Suppression of immunity – *Arg2, Vsig4*.

Multiple drug resistance, detoxification – *Abca1, Abca13, Aldh1l1, Gstm3*.

Anti-apoptotic genes – *Cd5l, Tnfrsf13c*.

Cytoskeleton reorganization – *Myo1b, Pdlim4, Ttll2, Tubb1, Trpv4, Upk1b, Cobl, Cp, Dock10*.

BM reorganization, adhesion, mobility in tissues – *Pde4d, Prg4, Nrcam, Perp, Comp, Igsf3, Pvrl1, S100a14, Adamts2, Acpp, Fcna, Fblim1, Dsc2, Col6a2, Col3a1, Hepacam, Itga9, Mmp2, Ltbp1*.

Dispersal via blood flow, exit from blood vessels – *Selp, Lyve1, Cldn1*.

Angiogenesis – *Pdzd3 (Pdzk1)*.

Survival in tissues – *Wnk2, Gas6*.

Calcium metabolism – *Pdk4*.

In total, 54 genes fell into the “Cancer” category, based on the literature data.

### Genes related to the cAMP-dependent signaling pathway

**Figure 3:**
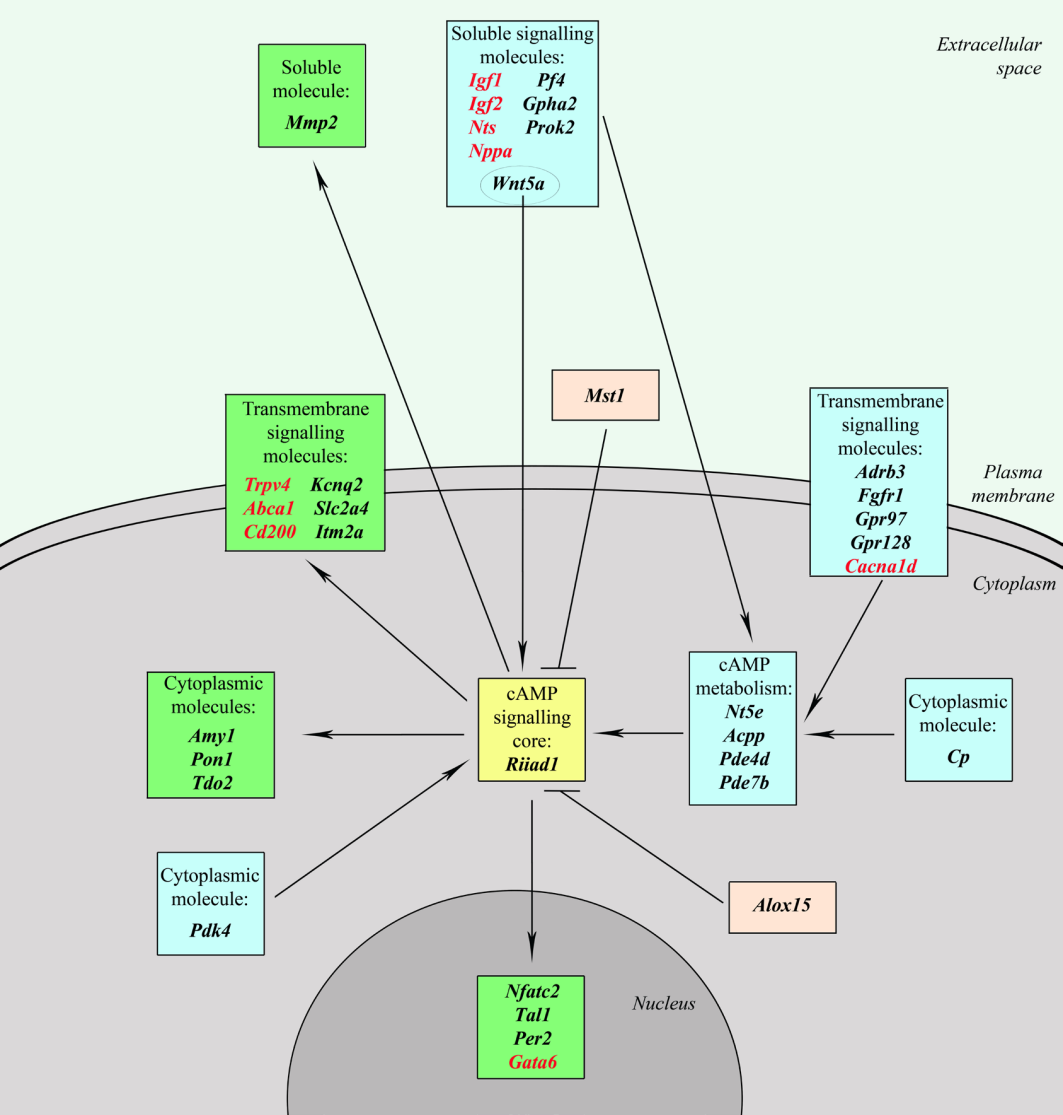
The cAMP-dependent signaling network Genes forming cAMP signaling core are shown in yellow; blue denotes upstream genes that regulate cAMP metabolism and activity of the signaling core; green – downstream genes that are affected by cAMP-signaling system; pink – genes which have an inhibitory effect on cAMP signaling pathway; genes that can form a positive feedback are shown in red.

Analysis of the genes differentially expressed in TAMRA+ cells has allowed us to identify a group of genes that one way or another are associated with the cAMP-dependent signaling pathway. This is one of the most ancient (if not the most ancient) signalling systems, which is broadly involved in a variety of cellular processes, such as cell survival and apoptosis evasion, immediate early response to mitogens, cell differentiation and dedifferentiation, metabolism of lipids and carbohydrates. The reconstructed network (Figure 3) includes more than 20% (36 out of 168) of the identified cancer stem cell-specific genes that, inter alia, provide a sufficient positive feedback to keep the system active (Supplementary Table S2): *Nt5e, Acpp, Pde4d, Pde7b, Igf1, Igf2, Pf4, Gpha2, Prok2, Nts, Wnt5a, Nppa, Adrb3, Fgfr1, Gpr97, Gpr128, Cacna1d, Cp, Pdk4, Riiad1, Trpv4, Abca1, Cd200, Gata6, Amy1, Nfatc2. Kcnq2, Itm2a, Pon1, Tal1, Per2, Slc2a4, Tdo2, Mmp2, Alox15, Mst1*.

All of the genes are conditionally divided into 3 groups: 1. upstream genes, that regulate cAMP metabolism and activity of the signaling core; 2. genes of the signaling system itself that include cAMP-metabolic enzymes and PKA complex; 3. downstream genes that are affected by the cAMP-signaling system. Genes with a positive feedback are shown in red. Most of the upstream genes affect the adenylate cyclase activity, but there are a number of them that affect the PKA and the related system itself. It is interesting to note that *Wnt5a* utilizes both ways: it triggers cAMP elevation at the plasma membrane and is implicated in increasing the catalytic subunits of PKA. Downstream genes, in turn, form 2 groups: 1) functionally activated ones that include transcription factors (like *Nfatc2* that is functionally activated by PKA-dependent glycogen synthase kinase-3β inactivation) or any cellular function effectors (like *Kcnq2* that is functionally activated by PKA-dependent phosphorylation) and 2) transcriptionally activated, which also include transcription factors (like *Per2*) and molecules that participate in some cellular process (like *Pon1*). Cellular processes, regulated by the downstream, include K+ (*Kcnq2*) or Ca2+ (*Trpv4*) transport, lipid (*Abca1*) and carbohydrate (*Amy1*) metabolism, as well as cell differentiation and commitment (*Itm2a*) and many others.

The remaining 55 genes – *Alas2, Amac1, Ankrd22, Ap1m2, Blnk, Catsperg2, Ccdc8, Ccr3, Cd55, Cplx1, Crabp2, Ddx3y, Dusp13, Fam107a, Fmnl2, Gdf6, Ggt7, Gng11, Gpr97, Gtpbp10, Hpn, Iglon5, Il10, Il17rb, Inhbe, Jsrp1, Lass4/Cers4, Lpcat2b, Maged2, Marco, Mycbpap, Nppa, Pdlim3, Ppfia4, Psd2, Pydc4, Pyroxd2, Rab15, Sec14l4, Sec31b, Sel1l3, Serpinb1a, Serpinb2, Slc40a1, Slc44a3, Slco4a1, Srms, Tdo2, Timp3, Tmem59l, Tmem82, Tnn, Upk3b, Vsig8, Xlr3a*, have also been annotated according to their functions described in the original publications, without binding them to the molecular systems determining the properties of TISCs. In this group, genes can be identified that participate in metabolic pathways as activators or repressors of molecular cascades of these pathways. These include growth factors (*Gdf6*, *Inhbe*), a kinase (*Srms*), a transcription factor (*Lass4*/*Cers4*), and cytokines (*Il10*, *Il17rb*). A number of these genes are insufficiently characterized in terms of their function and are of great interest as they may represent potential targets in anti-TISC therapy (at least in the context of Krebs-2 cancer cells).

It is to be noted that more than 40 genes characteristic for TAMRA+ TISC Krebs-2 (distributed across all the groups) are in the list of the core set of genes implicated in epithelial-mesenchymal transition of the cancer cells [3, 38] (Supplementary Table S2). The list includes two secreted trigger molecules – *Wnt5a* and *Igf* that are key to this process.

### Real Time PCR verification of differential gene expression data

**Figure 4:**
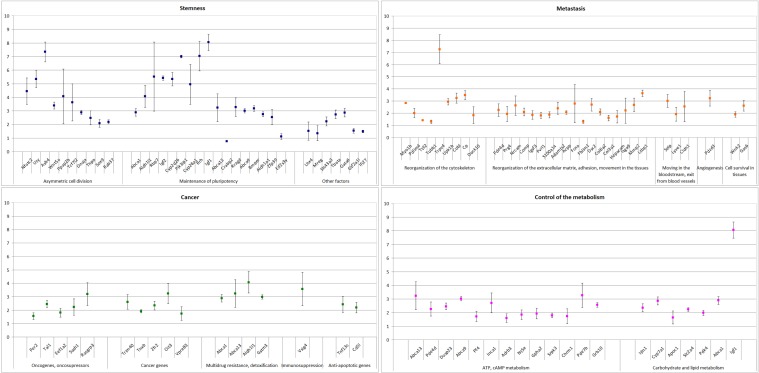
Real Time PCR validation of gene expression data of select genes identified in RNAseq The genes are split into main GO groups: stemness, cancer, metastasis, control of the metabolism.

To validate the results obtained in the RNAseq experiments, we performed qPCR on cDNA synthesized from polyA+ mRNA of TAMRA+ and TAMRA– cells. Expression of the main genes representative of the categories of interest was characterized. The results of this analysis are shown in Figure 4 and are represented as fold increase in expression in TAMRA+ cells vs TAMRA– cells.

**Figure 5:**
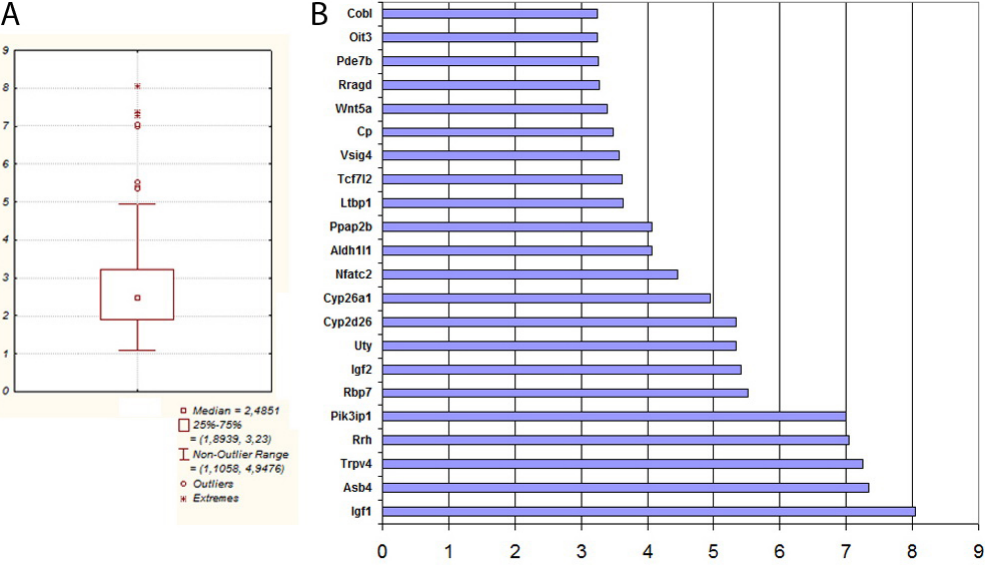
(**A**) Distribution of all gene expression of TAMRA+ Krebs-2 cells in qPCR. (**B**) List of 22 genes whose expression in TAMRA+ cells relative TAMRA– cells was maximal in qPCR.

The analysis performed confirmed the results of the RNAseq and allowed a group of genes to be identified that are overexpressed in *Krebs-2* cancer cells. In this group, two pairs of genes stand out: the secreted growth factor *Igf1* and the transcription factor *Nfatc2* activated by it, and cytokine *Wnt5* and its downstream target transcription factor *Tcf7L2* (Figure 5).

WNT5 is known to be a trigger molecule of the WNT5-dependent signaling pathway, while the transcription factor TCF712 activated as a result of triggering the WNT signaling cascade launches transcription of the genes of a genetic network determining the stemness properties of the TAMRA+ Krebs-2 cells [57–60]. In its turn, IGF2 is a trigger molecule of the MAPK signaling cascade, where the signaling converges on the transcription factor NFATC2 that induces transcription of the genes from a genetic network determining the cancer properties of cells [61–63]. At the same time, the list of over-expressing genes does not include any of the intermediate factors of the indicated signaling pathways. We believe TISCs may control the maintenance of their stem and cancer properties in an autocrine fashion, by permanently up-regulating the expression of these molecules. Secreted factors may ensure spatial protection from different extracellular regulatory molecules or other factors, in particular, regulatory exosomes. Increased local concentration of such factors may thus form a “protective shield” around a cell and maintains permanent extracellular trigger signaling [64–66]. High expression of specific transcription factors ensures mandatory activation of a strictly determined genetic network that shapes the phenotype of a TISC. It is noteworthy that NFATC2 activates the genes encoding secreted growth factors and cytokines (*Wnt5, Igf1, 2, Il-10*) and the genes of multifunctional transcription factors (*Gata6, Tal1, Tcf7L2*). Simultaneously, *Nfatc2* undergoes autoactivation (Supplementary Table S3). Such a pattern of interactions between the factors analyzed suggests that both pairs of proteins (IGF1-NFATC2, WNT5-TCF7L2), IL10, GATA6 and TAL1 are interconnected in the shared activation network and so they trigger and control several signaling cascades.

In the total list of the overexpressed genes, two groups are most noticeable, constituting more than half of the list and determining the stemness features of the cells – non-symmetric division and pluripotency. Increased expression of such genes may reflect the ontological necessity of the existence of this type of cells, primarily control and preserve the stemness properties, which provide a cell with a variety of advantages compared to a committed somatic cell.

### Comparative functional analysis of the genes specifically overexpressed in TAMRA+ Krebs-2 TISCs across database resources

Next, the gene set found was compared against current pathway/functional databases to evaluate their possible contribution to the regulation of Krebs-2 TISCs. To assess the functional associations, analysis was conducted using Ingenuity Pathway Analysis (IPA) and KEGG platforms that cluster the genes into functional groups, and using OPOSSUM system to analyze the potential targets of transcription factors.

**Figure 6:**
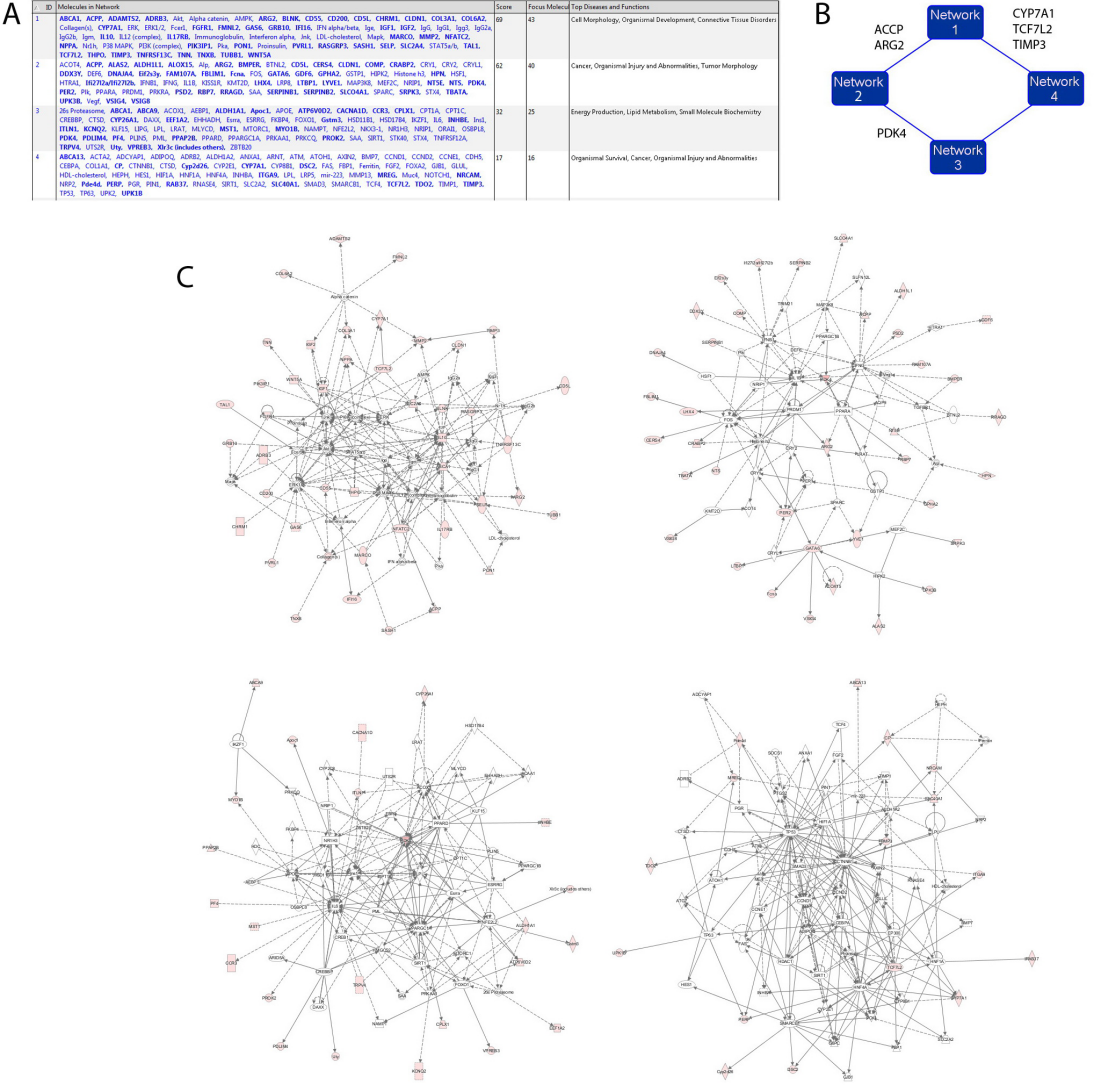
Hypothetical interaction networks of 168 genes expressed in TAMRA+ Krebs-2 TISCs as assessed by IPA (**A**) Cell functions in which genes overexpressed in TAMRA+ cells (RNAseq) participate. (**B**) 4 gene networks and functional molecules uniting them (a schematic view). (**C**) zoom-in of the 4 main gene networks.

When fed with 168 genes overexpressed in TAMRA+ Krebs-2 TISCs, IPA grouped them into 4 main genetic networks (Figure 6). As expected, in agreement with the manual analysis discussed above, these genes are primarily involved in cell functions such as organism development, cell morphology, and contribute to various molecular systems determining the biological properties of a cancer cell.

The four identified gene networks are interconnected by functional molecules (kinase PDK4, phosphatase ACPP, and the transcription factor TCF7L2) and so they may constitute a common system of functional associations in TAMRA+ Krebs-2 TISCs (Figure 6B).

In the total list of genes processed by IPA, groups of genes were identified that mediate, trigger, or block certain gene programs. The following categories were found: transcription factors, kinases, phosphatases, growth factors, and cytokines (Table 1).

**Table 1:** Groups of genes involved in triggering or blocking of certain gene programs

Kinases	Phosphatases	Growth factors	Cytokines	Transcription factors
*Fgfr1*	*Acpp*	*Clec11a*	*Il10*	*Gata6*
*Pdk4*	*Dusp23*	*Gas6*	*Pf4*	*Lass4*
*Srms*	*Nt5e*	*Gdf6*	*Thpo*	*Lhx4*
*Srk3*	*Pon1*	*Igf1*	*Wnt5a*	*Nfatc2*
	*Ppap2b*	*Igf2*		*Per2*
		*Inhbe*		*Tal1*
		*Mst1*		*Tcf7l2*
				*Sox9*

It turned out that the gene networks built by IPA encompass general cellular transcription regulators (*Nfatc2, Tal1, Gata6, Tcf7l2+*) as well as growth factors and kinases, that are known as key inducers of many important cellular signaling systems (*Fgfr1, Igf1,2, Mst1, Wnt5*).

OPOSSUM analysis produced a list of gene targets of *Nfatc2, Sox9, Tal1/Gata1, Tal1/Tcf3*, and *Tcf7l2+* transcription factors. This analysis suggests 69 genes out of 168 are under control of these factors, including own genes and the genes of other transcription factors (Supplementary Table S3). Noteworthy is activation by regulating the genes of the growth factors and cytokines, determining the specific functions of a cell and/or participating in maintenance of these functions (*Igf1,2, Mst1, Il10, Wnt5a, Gdf6*), which, in their turn, activate the said transcription factors, forming a loop of the function activation.

KEGG analysis indicates that several critical signaling pathways may be active in the TAMRA+ Krebs-2 TISCs. These signaling systems determine both stem and cancer phenotypes of these cells. Two gene pairs, *Igf2-Nfatc2* and *Wnt5-Tcf7l2+*, which are the main players in MAPK- and WNT-dependent signaling systems, were again revealed. The first gene pair is known to be activated in cancer cells, while the second one determines the stem properties of a cell. In addition, NFAT2C is the transcription factor of such signaling cell systems as cAMP and VEGF. One can also mention yet another interesting pair of up-regulated genes in TAMRA+ Krebs-2 TISCs that are involved in mTOR-dependent signaling pathway – these are *Igf1,2* and *Eef1a2*. That activity of this gene cascade is increased in Krebs-2 TISCs is supported by the fact the largest fold difference in expression of *Igf1,2* genes in TAMRA+ vs TAMRA– cells. As noted previously, in TAMRA+ Krebs-2 TISCs, activation of genes that encode other growth factors/cytokines and transcription factors and that do not appear to be involved in a common pathway, is detected by KEGG analysis. These include *Mst1, Il10*, *Gata6*, and *Sox9* genes, which are the functional components of the cGTP-, cAMP-, and FOXO-dependent signaling cascades.

## DISCUSSION

Since their discovery, TISCs have been actively investigated in many laboratories of the world. In addition to the importance of academic knowledge of the specific features of this type of cells, scientists are interested in the search for molecular factors distinguishing these cells from normal stem cells and from committed cells. It is assumed that, knowing certain discriminating molecules that are on top of signaling cascades determining the properties of TISCs, one can target and block these factors, thus destroying the signaling pathways and eliminating the cells from the pathological tissue, improving the therapeutic prognosis [67–69]. Currently investigations are being conducted of the molecular pattern of RNA expression of genes of different types of cells, including TISCs, in the hope to determine either the molecular target common for all the TISCs types or the molecular factor specific for only this type of TISC. To make a TISC preparation, either their ability to efflux lipophilic dyes, such as rhodamine or Hoechst, or fluorescent antibodies for a characteristic surface marker are used.

The TISC status is known to be determined by the activity of the genetic platforms activated by functionally tied signaling cascades forming stem and cancer properties of these cells. The main molecules of any characterized signaling pathways are represented by a functional pair – an extracellular trigger ligand, to which primarily secreted growth factors and cytokines (and their receptors) belong, and specific transcription factors activated by them, which trigger a gene platform, which determines the specific properties or the function of a cell. Other intermediate and finite factors mostly determine signal transduction, or connection with the other molecular cascades or the specific functional response of the cell.

Analysis of the genes overexpressed in the TAMRA+ cells has allowed us to propose the idea of autocrine control of the main qualities of TISCs due to increased expression of the specific trigger secreted factors launching signaling cascades, which determine the stem and cancer properties of TISCs and manifesting themselves in activation of the specific genetic platforms by ontologically related transcription factors, also characterized by increased expression.

We assumed this property to be characteristic of other types of TISCs. We analyzed the set of genes obtained in microarrays and by RNAseq analysis of 4 transcriptome libraries of TISCs (Table 2, Supplementary Table S2).

**Table 2:** Comparative analysis of the functional groups of genes in transcriptome libraries obtained for cancer stem cells of several types of cancer

	Cancer stem cells	Triggering extracellular ligands	Transcription factors	Activated signaling pathways
This study	Murine Krebs-2 tumor	*Gdf6*	*Nfatc2*	MAPK
		*Igf 1,2*	*Tal1*	WNT
		*Il10*	*Gata6*	mTOR
		*Mst1*	*Tcf7l2*	FOXO
		*Wnt5a*	*Sox9*	cGTP
				cAMP
				VEGF
[9]	Human liver carcinoma	*IGF BP5*	*FOSL1*	MAPK/ERK
				mTOR
				FOXO
[12]	Human ovarian adenocarcinoma	*EGFR*	*NFRB*	MAPK/ERK
		*FZD5, 7, 8*	*TLE*	WNT
		*HB-EGF*	*NFAT5*	PI3K/AKT/mTOR
		*IL6*	*RA/UNX*	FOXO
		*LTBP1*	*NKX3.1*	CSF1
		*LIF*	*NR4a2*	TGFβ1
		*NRG1*	*PLAGL2*	VEGF
		*NRP2*		EGF
				Notch
[84]	Human prostate cancer	*CSF2*	*STAT1*	MAPK/ERK
		*IFNK u IFNGR/1*	*NFKB1*	WNT
		*IL6*	*BTG1*	AKT/mTOR
		*PAPPA*		JAK/STAT
		*WNT5A*		NFKB
				PAPPA
[10]	A line of human osteosarcoma cells	*IGF1*	*NFIX*	MAPK
		*IHH*	*NFYB*	WNT
			*SPI1*	mTOR
			*TFDP1*	FOXO
				IGF

A gene library [9] was obtained for the cancer stem cells of the human liver carcinoma. For this type of cancer stem cells, the following ontological paired trigger molecules and TF are detected: extracellular activator/stabilizer IGF/IGFBP5 as a marker of the extracellular secreted growth factor [70] and the transcription factor FOSL1 [71], activating the specific gene platform and, in particular, triggering the synthesis of MMP9, which leads to reorganization of the extracellular matrix. IGF/IGFBP5 is a component of FOXO, mTOR and MAPK/ERK signaling cascades, FOSL1 is in the system of the mTOR and MAPK/ERK signaling pathways [72]. Both factors are responsible for activation of the gene network characterizing the cancer properties of a cell. Activity of an ontological pair determines cell invasion by remodeling the structures of the extracellular matrix, which is one of the characteristics of TISCs. In this type of cells, activated genes are associated with such biological functions as multiple drug resistance, fat metabolism, angiogenesis, proliferation, transport, response to inflammation, production of cytokines, a cell cycle, and signal transduction. Observed are suppression of the expression of genes involved in development of organs, response to hypoxia, fixation of nucleotides, translation and elongation, and hemotaxis.

The SP OCC transcriptome (the side population cell from an ovarian cancer cell line) was described in [12]. This list includes the transcripts of 5 main secreted factors, which participate in biological cellular processes determining the cancer properties of cells. These are *LTBP1*, *LIF*, *NRG1*, *HB-EGF*, *IL6*. Transcription of these genes suggests the active state of signaling pathways as PI3K/AKT [73], CSF1 [74], AKT/mTOR [75], TGFβ1 [76]. In addition, indicating the activity of the VEGF, EGF, WNT signaling cascades is expression of the genes of the respective receptors NRP2 [77], EGFR [78], FZD5, 7, 8 [79–82]. It is the FZD receptors that are required for maintaining the pluripotent state of the cells. Besides, the list of genes contains transcription factors connected with secreted molecules by functional ties. The NFRB, TLE, NFAT5, RA\UNX, NKX3.1, NR4a2 transcription factors are identified, which are active molecules of the FOXO-, ERK-, MAPK-, WNT-dependent signaling pathways. The PLAGL2 transcription factor is present, an expressed suppressor of differentiation of stem cells and of stem cancer glial cells. At the same time, PLAGL2 determines their ability of self-renewal expressed through modulation of the WNT/catenin-dependent signaling pathway [83]. It was also shown that the set of genes characterizing the activity of the NOTCH cascade is at a high level of expression. The activity of this signaling pathway also determines the ability of self-renewal as one of the characteristics of stemness of this type of cells. Thus, trigger secreted molecules and transcription factors under their control, which determine the stem and cancer properties of cells, are the present in the SP OCC transcriptome, too.

In total, genes were clusterized characteristic of such biological processes as the cell cycle, apoptosis, proliferation, transport, signal transduction, transcription and translation, metabolism and modification of protein in the type of cells analyzed. Such a set of activities characterizes high metabolic activity of cells, which is a characteristic feature of cancer cells.

Birnie and colleagues evaluated the stem and cancer cell expression profile for the human prostate cancer [84]. Despite the small number of the characterized genes, secreted trigger molecules and ontological transcription factors, determining activation of the specific gene platforms, are also identified in the transcriptome obtained. Secreted factors are represented by the *WNT5A, IL6, CSF2, IFNK u IFNGR/1*, and PAPPA genes [85–88]. There are two transcription factors STAT1 and NFKB1 and the modulator of many transcription factors BTG1 in this list [89–91]. The following ontological functional ties can be seen between the factors. Secreted IL6, IFNK and IFNGR/1 cytokines activate two main signaling pathways JAK/STAT and MAPK and are connected with the classical ontological partner STAT1. Phosphorylation of STAT1 is accompanied by transcription of the platform of the IF/IL6-induced genes, which characterizes development of an immune response. In addition, activation of JAK/STAT and MAPK results in activation of genes related to metastasizing, apoptosis, and proliferation, indicating participation of an ontological group in functioning of the cancer cell [86, 88, 89, 92, 93]. PAPPA and WNT5 are connected with the NFKB1 transcription factor by ontological ties. Paracrine activation of the NFKB-dependent signaling pathway may be accompanied both by an immune response (WNT5 [60]) and changes in signaling cascades determining cell survival and maintenance of the stability of its genome (PAPPA [87]). WNT5, as a trigger factor, directly activates the WNT-dependent signaling pathway determining one of the main properties of the stem cells, non-symmetric division. CSF2–GM-CSF is one of the factors required for proliferation, differentiation and survival of macrophages and granulocytes. In addition, its participation in the early embryo development in the sustenance of gestation has also been demonstrated [94–96], which may characterize the stem properties of the cancer cells of the human prostate cancer. CSF2 activates AKT1-, ERK1/2-, mTOR- but not JAK/STAT-dependent signaling cascades, which, as mentioned previously, are active in many cancer stem cells. Thus, the transcriptome analysis of the cancer stem cells of the human prostate cancer is in agreement with the idea that cancer stem cells are expressed by a small set of trigger molecules and by the transcription factors connected with them by functional ties, which maintain and protect their stem and cancer properties from the impact of the surrounding factors.

The combined analysis in the KEGG system indicates the activity of the clusters of genes determining mobility of the caner stem cells of the human prostate cancer. The group of genes of focal adhesion and the genes coding the proteins destroying matrix-receptor interaction have been identified.

Using an orthotopic mouse model, the pattern of gene expression profiling of the TISCs isolated from human osteosarcoma cell lines has been analyzed [10]. The following secreted trigger molecules IGF1, IHH, and NFIX, NFYB, SPI1, TFDB1 transcription factors have been identified in the expression profile obtained. All these factors are connected in one regulatory cluster, in which both the stemness and “cancer” properties of cells are determined. IGF1-IGFR1 is required for expression of *IHH*, a member of the family of *HH* mammal genes. The IGF-dependent signaling pathway modulates the expression of *IHH*, resulting in the control over cell proliferation, the survival, differentiation, and production of matrix in the growth plate in the course of post-natal development [97]. At the same time, IHH is connected with the WNT-dependent signaling cascade and activates it, reducing the level of the WNT antagonists (LRP, SFRP -) [98, 99]. Abnormal activation of the IHH-dependent signaling pathway due to increased expression of IGF leads to development defects and transformation of stem cells in TISCs, resulting in cancer development. The IGF-dependent signaling pathway is a component of the FOXO-, mTOR- and MAPK-dependent signaling cascades, the impaired activity of which also results in the carcinogenic transformation in the stem cells. Thus, abnormal expression of two trigger molecules IGF/IHH may be one of the causes of cancer development and of maintenance of the cancer properties of TISCs [99, 100].

Ontological interactions among the transcription factors analyzed have been established. SPI1 (PU.1) is the key regulator of hematopoiesis. Reduction of the expression of this transcription factor results in development of acute myeloid leukemia [101]. It is functionally associated with IHH. Joint expression of SPI1 and IHH results in the appearance of self-renewing leukemic cancer stem cells erythroid progenitors are infected with viruses with subsequent occurrence of leukemia [102]. E2F1 induces expression of NFYB, which together with E2F1 regulates the expression of many target genes. Excessive expression of these genes is found in osteosarcoma and is associated with non-sensitivity to chemotherapy. The NFYB/E2F1 complex becomes connected with FOXO1/3 and is the factor of the FOXO- dependent signaling cascade, which is activated in the cells of different cancers [103]. Another transcription factor, NFIX, also physically interacts with FOXA1 and regulates functioning and survival of hematopoietic stem cells and is also in the chain of signal transduction of the FOXO-dependent signaling cascade [104, 105]. The TFDP1 transcription factor is required to control the performance of a cell cycle and expression of target genes. GATA1 and TAL1 regulate the expression of *TFDP1*. Amplification of this gene results in deregulation of the cell cycle and in cancer development [106, 107]. Thus, the ontological pair SPI1 and IHH controls the primary property of a TISCs – self-renewal. The complex of the NFYB/E2F1- FOXO1/3 – NFIX transcription factors and the TFDP1 transcription factor regulate such properties of a TISC as aberrant proliferation, drug resistance, and survival. The combined comparison in the KEGG system indicates that the stem cell genes of the human osteosarcoma are clustered in the groups characteristic of two biological processes, development of skeletal bones and mobility (migration). No relevant differences in the expression of the known markers of *Oct/Nanog* embryo stem cells have been found.

Analysis of 5 transcriptomes of cancer stem cells made indicates that 1) the genetic platforms of different types of cancer stem cells differ; 2) there is no equally proportional simultaneous expression of all the genes of a certain signaling pathway; 3) TISCs autocrinely controls constancy of its stem and cancer properties, maintaining a permanently high level of expression of secreted trigger molecules, which maintain the permanent extracellular trigger signal. The high expression of the specific transcription factors of the ontological partners of trigger molecules ensures mandatory activation of a strictly defined gene platform, which forms the specific properties of a TISCs.

An inference from the analysis made is the idea that, in order to destroy the basic properties of cancer stem cells, it is necessary to eliminate the secreted fraction of the trigger molecules and of their ontological transcription partners immediately and simultaneously and to stop expression of the genes of all the participants of the manifested factors which determine “stemness’ and “cancer” features of this type of TISCs.

## MATERIALS AND METHODS

### Isolation of Krebs-2 ascites cells internalizing TAMRA-labeled DNA

*Alu*-TAMRA DNA is a 500 bp fragment of the human *Alu* repeat labeled with dUTP-5′-TAMRA by PCR [16]. Krebs-2 cells were incubated with *Alu*-TAMRA DNA (0.5 μg per 1 million cells in 200 μl RPMI-1640 medium) for 1 hour at room temperature. Separation of TAMRA+ and TAMRA– subpopulations was performed on a BD FACSAria cell sorter equipped with 100 mkm nozzle at the event rate varying between 2000 to 3500 cells. To detect TAMRA fluorescence, a 488 nm laser and a 585/42 channel (also known as a PE channel) were used.

Several rounds of flow cytometry-based cell sorting were used to obtain enough amounts of TAMRA+ cells. Due to the low content of TAMRA+ cells in the total Krebs-2 population (typically below 5%), the isolation of a reasonable number of pure TAMRA+ cells was nearly impossible. The best result we managed to achieve was 80% of TAMRA+ cells after a sorting pass. Subjecting the cells to multiple rounds of re-sorting resulted in drastic reduction in cell viability. So our population subsequently named as “TAMRA+ cells” consist of 60–70% of TAMRA+ cells and 30–40% of TAMRA– cells. In contrast to TAMRA+ cells, the pure TAMRA– subpopulation was virtually unlimited.

After sorting, microscopy analysis of the percentage of TAMRA+ cells was performed using a laser scanning microscope LSM 780 NLO (Carl Zeiss, USA) in the collective use center for microscopy of biological objects, Siberian Branch of the Russian Academy of Sciences.

### RNA isolation and RNAseq

Total RNA from TAMRA+ and TAMRA– cells was isolated using a Qiagen RNeasy kit. Libraries were constructed using Illumina TruSeq RNA sample preparation kit, v.2. The libraries were loaded on Illumina Hiseq2000 using Truseq v.3 reagents. After sequencing, the reads were sorted according to their indices with the CASAVA 1.8.2. software package (laboratory of evolutionary genomics, department of bioengineering and bioinformatics, Lomonosov Moscow State University, Moscow). Two independent DNA sequencing repeats produced 2 million paired-end reads 70 bp each, that were treated as single-end reads for mapping purposes.

### Reads analysis and software

The obtained reads were trimmed out of adapter sequences using a Trimmomatic tool from the Usadel Lab (http://www.usadellab.org/cms/?page=trimmomatic) and further mapped to murine chromosomes (available at the UCSC Genome Bioinformatics: http://hgdownload.soe.ucsc.edu/goldenPath/mm10/chromosomes/). As a mapping tool, the Softberry's ReadsMap (Softberry.Inc) software [108] was used. The unique feature of this tool is that it maps a certain read to the appropriate contig (a chromosome in our case) by selecting the best alignment from the pool of those found. Combined with another unique feature that allowed us to determine the splicing sites with high accuracy and thereby to drastically minimize the number of read “tails” mismapped to introns (http://www.softberry.com/berry.phtml?topic=readsmap-i&group=help&subgroup=pipelines and http://www.softberry.com/berry.phtml?topic=rnaseq), the ReadsMap tool produces precise mapping profiles that reflect the real number of reads aligned to the given position in the genome and in fact can be considered as relevant to the gene expression level. The trimmed reads are available at the European Nucleotide Archive (ENA) (http://www.ebi.ac.uk/ena/data/view/PRJEB15164).

Read counts were smoothed and log2-scaled to facilitate visual analysis of genes having extremely high expression differences (10 times and more). As a visualization tool for comparing the profiles and testing their correspondence to the annotated genes (KnownGenes available at UCSC Genome Bioinformatics: http://hgdownload.soe.ucsc.edu/goldenPath/mm10/database/), the Softberry's Sequence Explorer v.2.2.18 (http://www.softberry.com/) was used.

### qPCR primer selection

Krebs-2 ascites tumor cells were sorted into TAMRA+ and TAMRA– subpopulations and RNA was isolated. CDNA was synthesized for each group using a GoScript^™^ Reverse Transcription System (Promega, USA). A pair of primers was selected for each gene, which corresponded to the area (exon) of the highest read density. The primers were tested for specificity using the total Krebs-2 cDNA as a template. Real-time qPCR was run on an Applied Biosystems instrument using the SYBR^®^ Green PCR Master Mix (Applied Biosystems, USA) reagent in three replicas. Murine β-actin gene was used as a reference.

## SUPPLEMENTARY MATERIALS TABLES








