# Supplementary material for: Gene expression profiling of tumor-initiating stem cells from mouse Krebs-2 carcinoma using a novel marker of poorly differentiated cells
Source: Oncotarget. 2016 Dec 23;8(6):9425–41. doi: 10.18632/oncotarget.14116 (PMC5354742; doi:10.18632/oncotarget.14116)
Supplement: Supplementary file 1 [file oncotarget-08-9425-s001.pdf]

Gene expression profiling of tumor-initiating stem cells from mouse Krebs-2 carcinoma using a novel marker of poorly differentiated cells

Supplementary Materials

Supplementary Table S1: The list of genes investigated. See Supplementary_Table_S1

Supplementary Table S2: Comparison of investigated genes with literature data. See Supplementary_Table_S2

Supplementary Table S3: The list of genes activated by respective transcription factors. See Supplementary_Table_S3
